# A Case of Liposarcoma With Peritonitis Due to Jejunal Perforation

**DOI:** 10.1080/1357714031000114183

**Published:** 2003-03

**Authors:** Hisashi Horiguchi, Miwa Matsui, Tatsuo Yamamoto, Ryosuke Mochizuki, Takeshi Uematsu, Masachika Fujiwara, Hirotaka Ohse

**Affiliations:** 1 Center for Medical Sciences Ibaraki Prefectural University of Health Sciences Ami, Ibaraki Inashiki 300-0394 Japan; 2 Department of Pathology Kensei General Hospital Ibaraki Iwase Japan; 3 University of Tsukuba Hospital Ibaraki Tsukuba Japan; 4 Department of Surgery Shioya General Hospital Tochigi Yaita Japan

## Abstract

A 21-year-old man, who had been treated for congenital dilatation of the bile duct 13 years previously, presented with an acute abdomen. The physical examination suggested peritonitis, and an emergent laparotomy was performed. A perforation was foundin the jejunum approximately 100 cm distal to the ligament of Treitz, followed by resection of a 60-cm jejunal segment. No tumorous lesions were found during the operation, and the resected jejunal segment showed only focal myxomatous thickening of the serosa. Despite intensive therapy, he died of uncontrollable septic shock 2 days after the operation. Unexpectedly, however, histological examination revealed a liposarcoma, showing an unclassifiable histology. From the distribution of the lesion and the histological findings, it is thought that a primary lesion was somewhere else, covered by severe adhesions due to the previous operation, and that the tumor cells spreading from it could have caused the jejunal perforation through vascular involvement. Although extremely rare, liposarcomas in the abdomen can cause intestinal perforation. It is important for both clinicians andpathologists to carefully investigate the cause of an unusual clinical presentation such as intestinal perforation.


*Sarcoma* (2003) 7, 29–33

Taylor & Francis healthsciences

**CASE REPORT**

### A case of liposarcoma with peritonitis due to jejunal perforation

HISASHI HORIGUCHI[1], MIWA MATSUI[1,2], TATSUO YAMAMOTO[3], RYOSUKE MOCHIZUKI[4], TAKESHI UEMATSU[4], MASACHIKA FUJIWARA[2] & HIROTAKA OHSE[1]

[1]*Center for Medical Sciences, Ibaraki Prefectural University of Health Sciences, Ami, Ibaraki, Japan,* [2]*Department of Pathology, Kensei General Hospital, Iwase, Ibaraki, Japan,* [3]*University of Tsukuba Hospital, Tsukuba, Ibaraki, Japan &* [4]*Department of Surgery, Shioya General Hospital, Yaita, Tochigi, Japan*

**Abstract**

A 21-year-old man, who had been treated for congenital dilatation of the bile duct 13 years previously, presented with an acute abdomen. The physical examination suggested peritonitis, and an emergent laparotomy was performed. A perforation was found in the jejunum approximately 100 cm distal to the ligament of Treitz, followed by resection of a 60-cm jejunal segment. No tumorous lesions were found during the operation, and the resected jejunal segment showed only focal myxomatous thickening of the serosa. Despite intensive therapy, he died of uncontrollable septic shock 2 days after the operation. Unexpectedly, however, histological examination revealed a liposarcoma, showing an unclassifiable histology. From the distribution of the lesion and the histological findings, it is thought that a primary lesion was somewhere else, covered by severe adhesions due to the previous operation, and that the tumor cells spreading from it could have caused the jejunal perforation through vascular involvement. Although extremely rare, liposarcomas in the abdomen can cause intestinal perforation. It is important for both clinicians and pathologists to carefully investigate the cause of an unusual clinical presentation such as intestinal perforation.

### Introduction

Liposarcoma is one of the most common soft tissue sarcomas in adults.[1,2] The major sites of liposarcoma occurrence are the extremities (especially the thighs) and the retroperitoneum, followed by the inguinal regions with much less frequency.[1,2] The first clinical manifestation of liposarcoma is usually an insidiously growing, ill-defined, deep-seated mass, especially when it occurs in the abdomen or the retroperitoneum.[1] Of course, liposarcomas can cause some symptoms when they involve neighboring structures. They rarely involve the small intestine or the intestinal mesentery, and rarely cause obstruction,[3,4] intussusception,[5] or symptoms mimicking prostatism[6] and acute appendicitis.[7] However, it is extremely rare for liposarcomas to cause intestinal perforation and peritonitis. To our knowledge, there is only one such case reported in the Medline database – a report whose title indicates that liposarcoma of the intestinal mesentery caused peritonitis (Orzechowski H, Wlodarczyk K, 1984, written in Polish, no abstract available). Herein, we report a case of liposarcoma that caused peritonitis due to jejunal perforation.

### Clinical summary

The 21-year-old male patient had a history of previous operation for congenital dilatation of the bile duct. Although precise information could not be obtained, his family denied major problems associated with complications after the operation. The patient had been complaining for 3 months of appetite loss. He was carefully examined at a hospital, but the cause remained unknown. Thereafter, he abruptly presented with continuous abdominal pain that became progressively more severe. Upon admission the next day, his level of consciousness had decreased slightly. Since physical examination suggested peritonitis, an emergent laparotomy was performed. A moderate amount of purulent ascites had collected in the abdominal cavity. The small intestines, especially in the upper abdomen, were adhered to each other, probably due to the previous operation. A perforation site was found in the jejunum approximately 100 cm distal from the ligament of Treitz, and a 60-cm long jejunal segment was resected. The suspected cause of the perforation was adhesions due to the previous operation, because

Correspondence to: Hisashi Horiguchi, Center for Medical Sciences, Ibaraki Prefectural University of Health Sciences, Ami, Inashiki, Ibaraki, 300-0394, Japan. Tel.: +81-298-40-2213; Fax: +81-298-40-2313; E-mail: horiguchi@ipu.ac.jp

ISSN 1357-714X print/ISSN 1369-1643 online/03/010029–05 © 2003 Taylor & Francis Ltd
DOI: 10.1080/1357714031000114183

there were no lesions that would cause obstruction of the gastrointestinal tract. Despite intensive therapy, the patient died of uncontrollable septic shock 2 days after the laparotomy. An autopsy could not be performed.

### Pathological findings

Grossly, the mucosal surface of the resected jejunum was principally unremarkable except for perforation (Fig. 1a), while the serosal surface was mostly covered by purulent and fibrinous material. On cross-sections, the serosa was focally and irregularly thickened, and small hematomas were scattered around the perforation (Fig. 1b), spanning from 5 cm on its oral side to 15 cm on its anal side. The thickened areas and hematomas were mainly distributed on the mesenteric side. At the perforation site, the serosa of the anal side revealed marked myxomatous thickening (Fig. 1c).

Histologically, the thickened areas of the serosa were myxomatous, edematous, or focally fibrous,

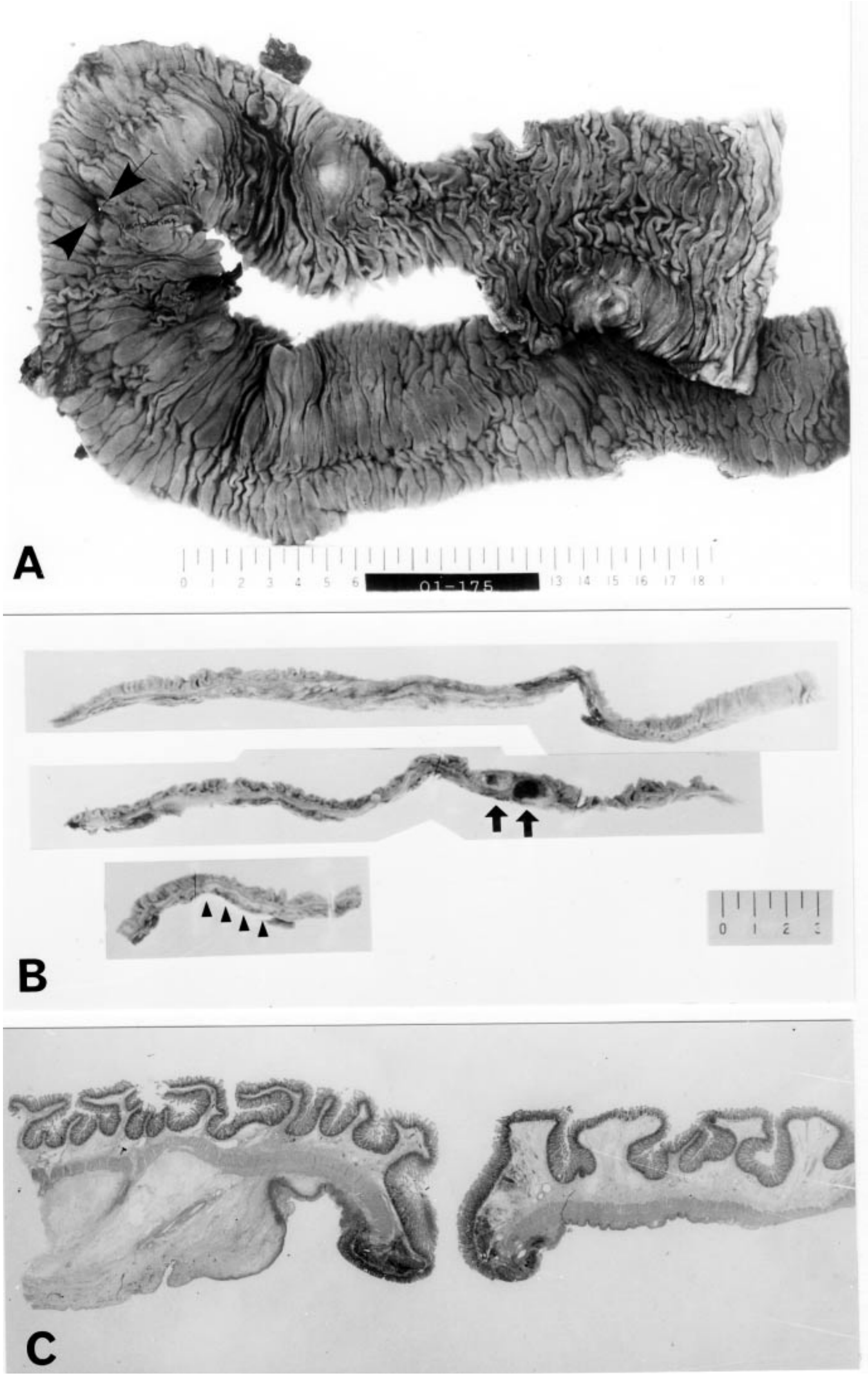

**Fig. 1.** *Macroscopic appearance of the resected jejunum. (A) There is a punched-out perforation (between the arrowheads), otherwise the mucosal surface is fairly unremarkable. (B) Cut strips reveal focal myxomatous thickening of the serosa (black triangles) and scattered hematomas (arrows). (C) Prominent myxomatous thickening of the serosa is seen on the anal side of the perforation site (left). (H&E, × 1).*

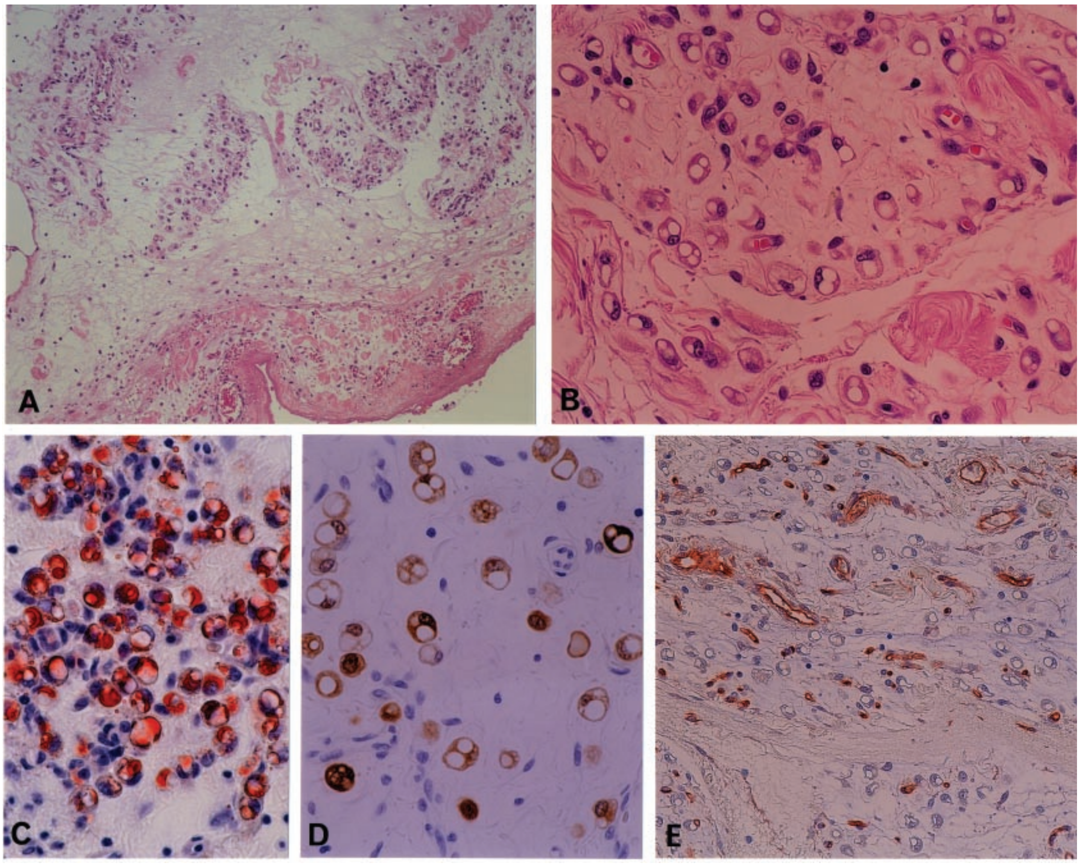

**Fig. 2.** *Histology and immunohistochemistry of the lesion. (a) The thickened serosa is myxomatous and edematous, and contains lobular structures. The serosal surface is covered by fibrinous exudates (lower right) (H&E, × 25). (b) Most tumor cells possess vacuolated cytoplasm, but a few show a short-spindled or naked appearance (H&E, × 100). (c) The cytoplasmic vacuoles are stained with oil red O on the cryostat sections of the formalin-fixed tissue ( × 100). (d) Immunohistochemically, tumor cells express S-100 protein ( × 100). (e) CD34 immunostaining is negative for atypical lipoblasts, and reveals no plexiform vascular pattern ( × 50).*

and contained lobular structures (Fig. 2a). The lobular structures appeared to spread along the vascular tree. In the lobules, round to oval cells loosely and separately proliferated in a myxoid matrix. These cells were also seen around hematomas. Most of the cells possessed abundant cytoplasm that contained one or a few vacuoles, whereas some cells had scant cytoplasm and showed a short-spindled or naked appearance (Fig. 2b). Their nuclei were mostly oval, showed coarse chromatin, and contained one or two nucleoli. Mitotic figures were not evident. The cytoplasmic vacuoles were clear and sharply delimited, and they distorted or displaced the nucleus to the side of the cell. The vacuoles were stained with oil red O (Fig. 2c), but not with alcian blue or periodic acid Schiff's reaction. The cells were also seen in the mesenteric fragments resected with the jejunum, but their infiltration into the muscular and mucosal layers was not observed. The serosal surface was covered with purulent exudates in which fungal hyphae and bacterial colonies were observed.

Immunohistochemically, tumor cells were positive for S-100 protein (Fig. 2d) and vimentin, but negative for CD68, CD34, and muscle specific actin. Though tumor lobules were relatively enriched with capillaries or small vessels, there was no plexiform vascular pattern, as noted upon CD34 immunostaining (Fig. 2e).

### RT-PCR analysis of TLS-CHOP and EWS-CHOP

The determination of the histological subtypes is important for prognosis and selecting therapy given that the clinical behavior of liposarcomas is closely related to their histological appearance.[1,2] To positively determine that the present case was an 'atypical' myxoid/round cell liposarcoma or not, we examined the expression of the TLS- or EWS-CHOP fusion transcripts by reverse transcription-polymerase chain reaction (RT-PCR) using paraffin-embedded material.[8]

Only routinely processed paraffin-embedded materials were available, thus limiting mRNA preservation and making it more difficult to detect longer transcripts.[9–12] However, it has been reported that there is much variation in the translocation points between the TLS and CHOP genes.[13,14] In addition, EWS-CHOP fusion transcripts are expressed instead of TLS-CHOP in a small number of cases.[15–17] Therefore, we designed several primer sets for the TLS gene, TLS-CHOP and EWS-CHOP fusion transcripts (Fig. 3). Extraction

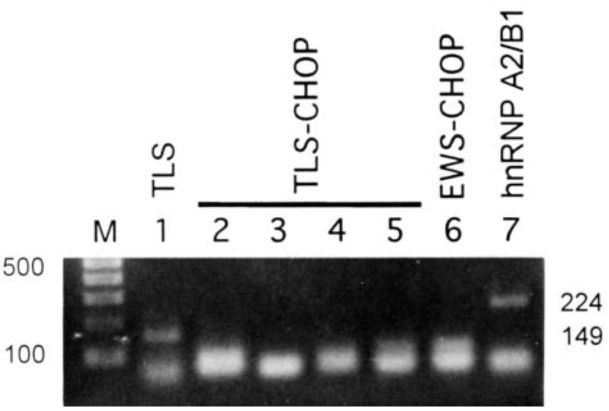

**Fig. 3.** *RT-PCR analysis of the lesion. TLS- or EWS-CHOP fusion transcripts were not detected (lanes 2–6), though TLS and hnRNP A2/B1 gene transcripts were amplified in the samples (lanes 1 and 7, respectively). The used reverse primer for the fusion transcripts is CHOP-R from lanes 2–6, and the used forward primers are as follows: lane 2, TLS-Ex3-F; lane 3, TLS-Ex5-F; lane 4, TLS-Ex7-F; lane 5, TLS-Ex13-F; lane 6, EWS-Ex7-F. M, size marker (100-bp DNA ladder). The amplification profile of the PCR consisted of 45 cycles of denaturation at 94°C for 50 s, annealing at 59°C for 45 s, and extension at 72°C for 1 min. The primer sequences are as follows: TLS-Ex1-F (5′-GCCTCAAACGATTATACCCA-3′); TLS-Ex3-R (5′-CCATAGCCTGAAGTGTCCGT-3′); TLS-Ex3-F (5′-CACGGACACTTCAGGCTATGG-3′); TLS-Ex5-F (5′-GTAGCAGTTCTCAGAGCAGCA-3′); TLS-Ex7-F (5′-ACCGTGGTGGCTTCAATAAAT-3′); TLS-Ex13-F (5′-GAATGAATGCAACCAGTGTAA-3′); EWS-Ex7-F (5′-CCAAGTCAATATAGCCAACAG-3′); CHOP-R (5′-CAGGTGTGGTGATGTATGAAG-3′); A2/B1-F (5′-CACCTTAGAGATTACTTTGAG-3′); and A2/B1-R (5′-CTCCTAGAACTCTGAACTTCC-3′).*

of total RNA and reverse transcription were performed using a Paraffin Block RNA Isolation kit (Ambion, Austin, TX, USA) and the PCR was carried out as described previously.[18]

The transcript of the hnRNP A2/B1 gene as a positive control for the integrity of mRNA in the sample could be amplified (Fig. 3). Using the TLS-Ex1-F and TLS-Ex3-R primer pair, 149-bp PCR products were also detected, and their DNA sequence analyses confirmed that the amplified fragments corresponded to the TLS transcript (data not shown). However, TLS- or EWS-CHOP fusion transcripts were not detected at all despite the fact that several repeat tests were performed.

### Discussion

The cause of jejunal perforation in the present case was at first misinterpreted as secondary to adhesions due to the previous operation, which had been performed for congenital dilatation of the bile duct 13 years previously. Histological examination of the resected specimen, however, revealed a liposarcoma. Although it is known that the bile duct and gallbladder carcinomas are associated with congenital dilatation of the bile duct,[19] such an association has never been reported for liposarcomas. Thus, it seems that there is no etiological relationship between a liposarcoma and the congenital disease in the present case.

Histologically, atypical lipoblasts with a myxoid matrix proliferated in a lobular fashion, looking like myxoid liposarcoma at a glance. However, the atypical lipoblasts were larger and more pleomorphic than those in classic myxoid liposarcomas. In addition, it lacked a delicate plexiform capillary pattern or hypocellular zones around the small arteries, which are among the characteristics of myxoid liposarcomas.[1,2,20] On the other hand, mature fat cells, which are seen in well-differentiated liposarcomas, were completely absent, and the degree of cellularity and pleomorphism were inappropriate for a pleomorphic liposarcoma. In accordance with a recent description,[2] we regarded the lesion as an unclassified liposarcoma. The negative results of RT-PCR analysis for TLS- or EWS-CHOP fusion transcripts seemed to support the diagnosis.[17,20]

Why was this patient's liposarcoma missed during the laparotomy? We think that the reason is primarily due to the unusual clinical presentation and the anatomical location. First, the patient presented with peritonitis due to jejunal perforation at admission. Liposarcomas rarely involve the small intestine (either as primary lesions or metastases) and cause some symptoms.[3–7] However, it is extremely rare for liposarcomas to cause intestinal perforation and peritonitis. Second, no tumorous lesions were found around the perforation site, although liposarcomas in the abdomen or retroperitoneum grow into large, sometimes huge and, as a rule, well-demarcated masses.[1,2] In the present case, the radiological examination before the operation was insufficient because of the emergent conditions preventing extensive evaluation. During the laparotomy, severe adhesions in the upper abdomen due to the previous operation might also have prevented more refined observations. In addition, the history of the previous operation probably influenced the clinical judgment as to the cause of the perforation. Histologically, the lobular structures of tumor cells were mainly scattered on the mesenteric side of the serosa, and they also existed in the mesenteric fragments. These findings suggest the possibility that tumor cells spread from the more proximal area of the mesentery, because lobular extensions of liposarcomas are not infrequently seen in the periphery of or apart from the main tumor.[1] Therefore, we think that a primary tumor may exist somewhere, probably near the root of the mesentery, covered by severe adhesions, and that the tumor cells observed in the resected specimen had spread from it, although this could not be confirmed by autopsy.

The cause of the jejunal perforation was not due to direct infiltration of tumor cells, because tumor cells were not observed at the perforation site. However, the perforation occurred at the boundary of the region with tumor cell infiltration, which showed marked myxomatous thickening of the serosa (Fig. 1c).

Histologically, tumor cells mostly spread along the vascular tree with a myxoid matrix. These findings suggest that the perforation was located at the most peripheral portion of a given vascular tree. Moreover, several hematomas along with a scattering of tumor cells were found in the serosa, indicating that tumor cells involved at least some vessels. Although a feeding artery could not be identified, it seems reasonable that the jejunal perforation would be caused by insufficient perfusion secondary to perivascular infiltration of the tumor cells with a myxoid matrix.

In summary, we have presented a case of liposarcoma with peritonitis due to jejunal perforation. Although no tumorous lesions were found during emergent laparotomy, histological examination revealed a liposarcoma. From the distribution of tumor cells and the histological findings, it is thought that a primary lesion exists somewhere, covered by severe adhesions due to the previous operation, and that the tumor cells spreading from it could have caused the jejunal perforation through vascular involvement. Although extremely rare, liposarcomas in the abdomen can cause intestinal perforation. This case warns both clinicians and pathologists to carefully investigate the cause of an unusual clinical presentation such as intestinal perforation.

### Acknowledgements

The authors appreciate the valuable suggestions of Dr. Takesaburo Ogata (Tsukuba Gakuen Hospital, Emeritus Professor of the University of Tsukuba) and Dr. Takuya Yazawa (Yokohama City University School of Medicine). The authors also thank Mr. Tohru Hoshi and Mr. Mitsuhiko Ono (Shioya General Hospital) for their technical assistance.

### References

1. Enzinger FM, Weiss SW. Liposarcoma. In: *Soft tissue tumors*. 3rd ed. St. Louis, MO: Mosby, 1995: 431–66.
2. Kempson RL, Fletcher CDM, Evans HL, Hendrickson MR, Sibley RK. Lipomatous tumors. In: *Tumors of the soft tissues*. 3rd series. Washington, DC: Armed Forces Institute of Pathology, 2001: 187–238.
3. Papadopoulos T, Kirchner T, Bergmann M, Müller-Hermelink HK. Primary liposarcoma of the jejunum. *Pathol Res Pract* 1990; 186: 803–6.
4. Nagawa H, Tsuno N, Saito H, Muto T. Ileal obstruction due to metastatic liposarcoma: a case report. *Gastroenterol Jpn* 1993; 28: 706–11.
5. Liaw CC, Wang CS, Ng KK, Lin PY. Enteric intussusception due to metastatic intestinal tumors. *J Formos Med Assoc* 1997; 96: 125–8.
6. Moyana TN. Primary mesenteric liposarcoma. *Am J Gastroenterol* 1988; 83: 89–92.
7. Rivkind AI, Admon D, Yarom R, Schreiber L. Myxoid liposarcoma of the small intestine mimicking acute appendicitis. *Eur J Surg* 1994; 160: 251–2.
8. Hisaoka M, Tsuji S, Morimitsu Y, Hashimoto H, Shimajiri S, Komiya S, Ushijima M. Detection of TLS/FUS-CHOP fusion transcripts in myxoid and round cell liposarcomas by nested reverse transcription-polymerase chain reaction using archival paraffin-embedded tissues. *Diagn Mol Pathol* 1998; 7: 96–101.
9. Rupp GM, Locker J. Purification and analysis of RNA from paraffin-embedded tissues. *Biotechniques* 1988; 6: 56–60.
10. Koopmans M, Monroe SS, Coffield LM, Zaki SR. Optimization of extraction and PCR amplification of RNA extracts from paraffin-embedded tissue in different fixatives. *J Virol Methods* 1993; 43: 189–204.
11. Foss RD, Guha Thakurta N, Conran RM, Gutman P. Effects of fixative and fixation time on the extraction and polymerase chain reaction amplification of RNA from paraffin-embedded tissue. Comparison of two housekeeping gene mRNA controls. *Diagn Mol Pathol* 1994; 3: 148–55.
12. Inoue T, Nabeshima K, Kataoka H, Koono M. Feasibility of archival non-buffered formalin-fixed and paraffin-embedded tissues for PCR amplification: an analysis of resected gastric carcinoma. *Pathol Int* 1996; 46: 997–1004.
13. Crozat A, Åman P, Mandahl N, Ron D. Fusion of CHOP to a novel RNA-binding protein in human myxoid liposarcoma. *Nature* 1993; 363: 640–4.
14. Panagopoulos I, Mertens F, Isaksson M, Mandahl N. A novel FUS/CHOP chimera in myxoid liposarcoma. *Biochem Biophys Res Commun* 2000; 279: 838–45.
15. Dal Cin P, Sciot R, Panagopoulos I, Åman P, Samson I, Mandahl N, Mitelman F, Van den Berghe H, Fletcher CD. Additional evidence of a variant translocation t(12;22) with EWS/CHOP fusion in myxoid liposarcoma: clinicopathological features. *J Pathol* 1997; 182: 437–41.
16. Antonescu CR, Elahi A, Healey JH, Brennan MF, Lui MY, Lewis J, Jhanwar SC, Woodruff JM, Ladanyi M. Monoclonality of multifocal myxoid liposarcoma: confirmation by analysis of TLS-CHOP or EWS-CHOP rearrangements. *Clin Cancer Res* 2000; 6: 2788–93.
17. Antonescu CR, Elahi A, Humphrey M, Lui MY, Healey JH, Brennan MF, Woodruff JM, Jhanwar SC, Ladanyi M. Specificity of TLS-CHOP rearrangement for classic myxoid/round cell liposarcoma: absence in predominantly myxoid well-differentiated liposarcomas. *J Mol Diagn* 2000; 2: 132–8.
18. Horiguchi H, Sakane M, Matsui M, Wadano Y. Bizarre parosteal osteochondromatous proliferation (Nora's lesion) of the foot. *Pathol Int* 2001; 51: 816–23.
19. Albores-Saavedra J, Henson DE, Klimstra DS. *Tumors of the gallbladder, extrahepatic bile ducts, and ampulla of Vater*. 3rd series. Washington, DC: Armed Forces Institute of Pathology, 2000.
20. Orvieto E, Furlanetto A, Laurino L, Dei Tos AP. Myxoid and round cell liposarcoma: A spectrum of myxoid adipocytic neoplasia. *Semin Diagn Pathol* 2001; 18: 267–73.
